# Orthopoxvirus Genes That Mediate Disease Virulence and Host Tropism

**DOI:** 10.1155/2012/524743

**Published:** 2012-07-30

**Authors:** Sergei N. Shchelkunov

**Affiliations:** ^1^Department of Genomic Research, State Research Center of Virology and Biotechnology VECTOR, Koltsovo, Novosibirsk Region 630559, Russia; ^2^Institute of Cytology and Genetics, Siberian Branch of the Russian Academy of Sciences, Novosibirsk 630090, Russia

## Abstract

In the course of evolution, viruses have developed various molecular mechanisms to evade the defense reactions of the host organism. When understanding the mechanisms used by viruses to overcome manifold defense systems of the animal organism, represented by molecular factors and cells of the immune system, we would not only comprehend better but also discover new patterns of organization and function of these most important reactions directed against infectious agents. Here, study of the orthopoxviruses pathogenic for humans, such as variola (smallpox), monkeypox, cowpox, and vaccinia viruses, may be most important. Analysis of the experimental data, presented in this paper, allows to infer that variola virus and other orthopoxviruses possess an unexampled set of genes whose protein products efficiently modulate the manifold defense mechanisms of the host organisms compared with the viruses from other families.

## 1. Introduction

In the course of evolution, viruses have developed various molecular mechanisms allowing them to evade the host's defense reactions [1–3]. Viruses can become particularly dangerous when they evolve to acquire the possibility to infect new animal species [4, 5]. The defense systems of the new host may be generally unable to counteract the new pathogen and many individuals will die. In any epidemics, there are also individuals showing little sensitivity or complete resistance to the particular pathogen. Both increased sensitivity and resistance to the infection are specified by the individual's genetic makeup and various environmental factors. Accordingly, mass epidemics not only produce new virus variants but also alter the host population structure: highly sensitive individuals die, while the portion of resistant individuals in the population increases. Therefore, the coevolution of the virus and the host is a mutually dependent process. It should be noted that mutational frequencies that drive genetic variations in viruses are much higher than in mammals [6]. On the other hand, animal genomes contain incomparably higher numbers of genes, while virus resistance mutations usually affect one or, less frequently, several genes. Such mutations may affect virus adsorption on the target cells, its replication, and/or the evasion of the host's defense systems.

Poxviruses are the largest mammalian DNA viruses with the developmental cycle taking place in the cellular cytoplasm [7]. These viruses encode a large set of proteins providing for extranuclear synthesis of virus mRNAs, replication of virus DNA, and assembly of complex virions and are involved in the regulation of multifactorial interactions of the virus with both individual cells and infected host organism. The unique properties of poxviruses attract close attention of researchers. The viruses belonging to the genus *Orthopoxvirus* are best studied among other viruses of the family Poxviridae, because this genus includes four virus species pathogenic for humans: variola (smallpox) virus (VARV), monkeypox virus (MPXV), cowpox virus (CPXV), and vaccinia virus (VACV). These orthopoxviruses are immunologically cross-reactive and cross-protective, so that infection with any member of this genus provides protection against an infection with any other member [3]. An important experimental model is ectromelia virus (mousepox virus, ECTV) [8, 9].

VARV causes smallpox and is an exclusively anthroponotic agent. For years, this human pathogen caused epidemics of disease with mortalities of 10–40%. Only the coordinated efforts of the world community, under the aegis of the World Health Organization, accomplished the eradication of smallpox [3, 10]. 

Natural reservoir of MPXV is rodents. Human monkeypox resembles the clinical course of smallpox that was prevalent on the African continent and is recorded predominantly in Central and Western Africa [11, 12]. Its mortality rate in several studied human monkeypox outbreaks in Central Africa reached 16% [3, 11]. The specific feature of human monkeypox clinical course, distinguishing it from smallpox, is lymphadenitides. Another difference between the human monkeypox and smallpox is in that the human-to-human transmission efficiency of MPXV is considerably lower as compared with VARV [3]. That is why this virus has not so far caused any expanded epidemics. 

CPXV displays the widest host range among the orthopoxviruses. Generally, human cowpox is a benign disease manifesting itself by isolated local lesions [3]. Human cowpox is recorded in the majority of European countries. Rodents (the main natural reservoir) or home pets and cattle (bridging hosts) represent the main sources of human CPXV infection [13–15]. In immunocompromised persons cowpox virus can cause a generalized eruption [16, 17] with lethal outcome in some cases [18].

VACV, used for vaccinating humans against smallpox, can be transmitted to man accidentally by contact with a vaccinee. Last years the number of reported outbreaks of the human diseases caused by the zoonotic VACV-like viruses is increasing in several countries [19–21].

VARV infection is a rare example of a strict anthroponosis caused by a virus propagating and spreading only within human populations; it is highly pathogenic for humans, being well adapted to overcome the defense barriers of this particular host. MPXV, CPXV, and VACV are zoonotic viruses with a wide range of sensitive species; they are evolutionary adapted to propagate in different mammalian hosts. In humans, they cause relatively rare sporadic disease cases when the virus is transmitted from an affected animal to a human [3]. ECTV, similarly to VARV, has a very narrow host range, being highly pathogenic only for certain mouse strains [8]. 

It is believed that viruses during coevolution with the host organism had incorporated into their genomes the coding sequences of various cellular genes and modified them for adapting to provide for their viability and preservation in the biosphere [22, 23]. Acquiring the knowledge about how viruses overcome numerous protective systems of mammals, which are represented by molecular factors and cells of the immune system, we will not only get a deeper understanding but also discover new patterns in organization and functioning of these most important mammalian organism responses directed against infectious agents.

## 2. Suppression of Molecular Recognition of Viruses by Innate Immune Cells

Innate immune cells express a large repertoire of germ line-encoded pattern recognition receptors (PRRs) that recognize microbial components. The receptors include toll-like receptors (TLRs), nod-like receptors (NLRs), RIG-1-like receptors (RLRs), and AIM2-like receptors (ALRs) [24]. These PRRs bind microbial ligands and initiate signaling cascades which result in the activation of transcription factors such as nuclear factor kappa B (NF-*κ*B), interferon regulatory factors (IRFs), and activating protein-1 (AP-1) involved in the expression of inflammatory and type I interferon (IFN) genes [1].

In response to infection, cells constituting the mammalian innate immune system, such as macrophages and dendritic cells, produce proinflammatory cytokines. IL-1*β* and IL-18 are synthesized as cytoplasmic precursors, which should be cleaved by a cysteine protease termed caspase-1 to acquire the active form. Caspase-1, in turn, is also synthesized as an inactive precursor, which can be activated within a large cytosolic protein complex called inflammasome [25–27]. Inflammasomes act as intracellular sensors responsive to conserved microbial components similarly to the functioning of TLRs on the cell surface or in endosomes. Proteins of the TLR family possess an intracellular TIR domain, which responds to infection by triggering intracellular signal cascades that activate innate immune reactions.

It was revealed that orthopoxvirus genomes contain two genes of TIR-containing proteins: VACV A46 and A52 [28–30]. These proteins have different functions and specifically inhibit intracellular signal cascades activating transcription factor NF-*κ*B, critical for innate immunity. A46 interacts with the factors MyD88, TIRAP, TRAM, and TRIF, and A52 with IRAK2 and TRAF6. It should be noted that CPXV and some VACV strains encode both above proteins, while VARV and MPXV, which are most pathogenic for humans, do not produce A52 VACV isologs (Table 1).

The crystallographic structure of A52 has been solved showing that this one is homodimer with folding similarity to B-cell-lymphoma- (Bcl-) 2-like proteins whose members inhibit apoptosis or activation of proinflammatory transcription factors [31]. To date a set of Bcl-2-like orthopoxviral proteins was discovered and characterized [32, 33] (Table 1). While these proteins share structural similarity, their degree of amino acid similarity is low indicating that they diverged long ago and although they share an ability to manipulate innate immune signaling pathways, they differ in their targets (Table 1) and mechanisms of action [32]. 

The crystallographic structure of VACV protein N1 (see Table 1) identified a groove similar to those of cellular antiapoptotic Bcl-2 proteins. N1 is therefore unusual in its dual ability to modulate both apoptosis and inflammatory signaling. N1 inhibits proapoptotic and proinflammatory signaling using independent surfaces of the protein [34]. Analyses of the other available three-dimensional structures of the orthopoxviral Bcl-2-like proteins have shown that VACV proteins A52, B15, and K7 do not contain BH3-peptide-binding groove important for inhibition of apoptotic stimuli and these proteins inhibit only activation of proinflammatory transcription factors [31, 32].

Based on the sequence/structure similarity, it was proposed that additional VACV proteins N2, C1, and C16/B22 (and their orthopoxviral isologs) might have a similar role in suppression of PRR-induced host immune response as other studied Bcl-2-like proteins (Table 1), by antagonizing at different levels with the TLR signaling pathways [32, 34]. 

Thus, orthopoxviruses have a multigenic system controlling their recognition by innate immune cells. The detected distinctions between the Bcl-2-like genes of VARV, MPXV, CPXV, and VACV require further studies of the properties of the corresponding proteins.

## 3. The Ubiquitin-Proteasome Pathway in Viral Infection

It has been recently discovered that protein degradation is an especially important regulatory cell process [35, 36]. In the majority of cases, proteins in eukaryotic cells are degraded via an ubiquitin-directed pathway. Ubiquitin (Ub) comprises 76 amino acid (aa) residues and is among the most evolutionarily conserved polypeptides; Ub is covalently attached to target proteins by a coordinated action of three enzyme classes [37].

The ubiquitin-activating enzyme (E1) cleaves ATP to form a thioester bond between the Ub C-end and the cysteine in the active site of this enzyme. Thus, activated Ub is then transferred to the ubiquitin-conjugating enzyme (E2), also forming a thioester bond. E2–Ub interacts with ubiquitin-protein ligase (E3), which concurrently binds the target protein, frequently named the substrate. E3 causes transfer of the Ub from E2–Ub complex to the substrate by formation of the covalent isopeptide bond between the Ub C-end and the lysine residues in target protein (substrate). Attachment of a single Ub can change the function or localization of the protein in the cell. A tandem attachment of Ub molecules, producing a polyubiquitin chain, can also modify the function or cell localization of target protein or causes involvement of such protein in degradation by the cellular 26S proteasome leading to protein cleavage into short peptides and Ub release [35].

Ubiquitin is the first member of the ever increasing family of ubiquitin-like (Ubl) proteins, which are also involved in modification of various proteins and their functions. Such modification processes are frequently of transient character because of existence of Ub/Ubl-deconjugating enzymes (Ub/Ubl-specific proteases) along with Ub/Ubl-conjugating enzymes. It has been discovered that Ubl attachment to target protein can frequently enhance the interaction of this modified protein with other proteins or, on the contrary, block its interaction with the target [37].

The most numerous group of E3 ligases contains cullin-RING ubiquitin ligases (CRLs), multisubunit complexes comprising cullin proteins [38], RING H2 finger proteins (designated Rbx1, Roc1, or Hrt1) [39], variable substrate-recognition subunit (SRS), and, for the majority of CRLs, additional adaptor proteins uniting SRS with other CRL proteins [36].

**Figure 1:**
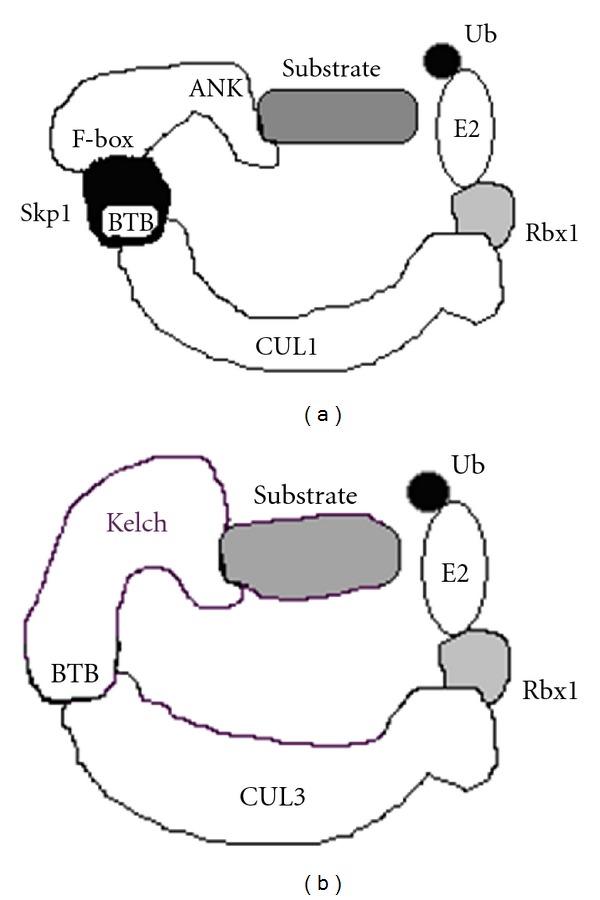
A schematic of two classes of orthopoxvirus E3 ubiquitin ligases: (a) SCF E3 ligase and (b) BTB-kelch/Cul3 E3 ligase.

Proteins of the cullin family are hydrophobic proteins playing the role of a backbone for assembly of the CRL complex [36, 38]. The CRL containing cullin-1 (CUL1), named SCF complex, has been most intensively studied. This complex comprises four subunits—Skp1, CUL1, F-box-containing protein, and Rbx1 (Figure 1(a)). The N-end of CUL1 protein binds to the Skp1 adaptor, which, in turn, interacts with F-box-containing protein. The C-terminal part of CUL1 binds to Rbx1 protein, whose function is in the interaction with E2-Ub. In turn, F-box-containing protein [40, 41] provides for the interaction with substrate protein, which is ubiquitinated by the complex (see Figure 1(a)).

The CUL3-containing CRL complexes contain Rbx1; however, they differ from the other studied CRL classes by the absence of adaptor proteins [42]. The BTB-domain-containing protein, accomplishing interaction with substrate protein of the complex by another protein-protein binding domain [43], directly interacts with the N-terminal CUL3 region (see Figure 1(b)).

Thus, the information accumulated so far demonstrates that a tremendous diversity of CRL complexes can be formed in mammalian cells. This agrees with the modern understanding that the modification of proteins by ubiquitin or ubiquitin-like polypeptides is important for the fate and functioning of the majority of proteins in eukaryotic cell and can be involved in regulation of various biological processes [44–46].

Taking into account the importance of ubiquitin-ligase and ubiquitin-proteasome systems for the function of eukaryotic cells, the role of viruses in regulation of these processes has been intensively studied recently. Although the data on this topic are sparse, it has been already discovered that viruses of various families can influence the protein ubiquitination to overcome the cell defense mechanisms, including apoptosis, type I interferon response, and antigen presentation by the class I major histocompatibility complex [45, 47, 48].

The developmental cycle of orthopoxviruses takes place in the cell cytoplasm. Orthopoxviruses replicate in the discrete cytoplasmic structures called virus factories or virosomes. These structures are encompassed by endoplasmic reticulum membranes, resembling cytoplasmic mininuclei [49].

Recent experiments with VACV have demonstrated that proteasome inhibitors interfere with formation of virus factories in the cytoplasm of permissive cells and, as a consequence, lead to a radical decrease in virus replication [50, 51]. These results suggest that a normal development of orthopoxvirus infection requires a functioning ubiquitin-proteasome system.

Since it has been discovered that ubiquitin constitutes at least 3% of the total protein in VACV virions [52], this suggests that either the ubiquitin-ligase system is important for modification of virus proteins and assembly of virus particles or ubiquitin-modified proteins are packaged into virions for further involvement in the early infection stages in sensitive cells.

Metabolism of animal viruses depending on their specific features is directed to utilize the structures of the cell cytoplasmic or nuclear protein skeleton. In particular, it is assumed that the virus-specific cytopathic effect is determined not by cell damage meaningless from the virus standpoint but rather by a specific rearrangement of cytoskeleton elements that would create the conditions for virus reproduction [53, 54]. The cytoskeleton proteins are encoded by a large set of various genes with a tissue-specific expression. This determines the difference between the compositions of protein “backbone” in different cell types, which influences the functions of these cells [55]. These differences can influence the parameter of virus replication in the host organism, such as tissue tropism. In addition, the protein composition of cytoskeleton in various mammalian species (or cell cultures) can influence the overall sensitivity to certain viruses and determine the so-called host range. It is known that individual orthopoxvirus species considerably differ in the range of animal species where they can reproduce [56].

### 3.1. Viral Ankyrin-F-Box Containing Proteins

It is known that viral infections activate the cell antiviral signaling and inflammatory responses. The nuclear factor NF-*κ*B, which regulates transcription of the genes involved in development of the apoptosis, inflammation, immune response, and cell proliferation [57], plays an important role in these responses. Activation of NF-*κ*B is controlled by ankyrin (ANK) repeat containing proteins of the I*κ*B family, which interact with this factor. In an inactive form, NF-*κ*B (the dimer p65/p50) is bound to the inhibitory protein I*κ*B*α* which via six ANK repeats interacts with p65 subunit. In response to molecular signals of infection, I*κ*B*α* kinase (IKK) is phosphorylated by cellular protein kinase to phosphorylate I*κ*B*α* at the serine residues at positions 32 and 36. The phosphorylated I*κ*B*α* is polyubiquinated by the SCF complex at lysine 48 and degraded by the 26S proteasome complex, thereby removing the NF-*κ*B inhibition; this factor moves to the cell nucleus and stimulates gene transcription via the interaction with specific DNA sequences [57, 58].

Different VACV strains inhibit activation of the cellular transcription factor NF-*κ*B, thereby providing inhibition of inflammatory response development, which is among the first reactions of nonspecific protection from infectious agents. It was demonstrated that the highly attenuated VACV strain MVA failed to inhibit NF-*κ*B activation. Recombination-based introduction of the *K1L* gene from VACV strain WR to the MVA genome restored the ability of the virus to inhibit the activation of cellular factor NF-*κ*B [59]. VACV protein K1 belongs to the family of ANK proteins [60–62] and it was assumed that K1 can inhibit degradation of the cellular I*κ*B*α* via competing with it for phosphorylation by the enzyme IKK and subsequent ubiquitination and degradation. Another ANK-containing protein, CPXV C9 (not synthesized by VACV; Table 1) is likely to act in an analogous manner [58] and, consequently, is able to rescue the mutation VACV *K1L* ^−^ [63].

Our analysis has demonstrated [62–64] that orthopoxviruses code for a large set of ANK proteins (Table 2). This is the largest family of orthopoxvirus proteins; moreover, each species has its specific set of the corresponding genes [63]. Of the natural orthopoxviruses, CPXV, displaying the widest host range, has 14 unique ANK genes in its genome (two of them are duplicated in the terminal genomic regions); MPXV, eight such genes and VARV, a stringently anthroponotic virus, five ANK genes, VACV-COP encodes five ANK proteins (three match the VARV proteins), while the highly attenuated variant VACV-MVA, obtained by multiple passages on chorioallantoic membranes (CAMs) of chick embryos and having a very narrow range of sensitive cell cultures, retained only one ANK gene [65].

It has been shown that many poxvirus ANK proteins contain F-box sequences at their C-ends [66]. Such combination of domains is characteristic of poxviruses only. In the cellular proteins, F-box domain is usually localized to the N-terminal part. In addition, the combination of ANK and F-box domains has not been found in cellular proteins [67, 68]. Our analysis allowed to detect F-box sequences in the C-terminal regions in 13 of the 14 CPXV ANK proteins (Table 1) [63].

Recently, it has been experimentally demonstrated for the ANK-F-box proteins C9 (CHOhr) of CPXV [58], G1 of VARV (D3/H3 in CPXV) [69], 186R of VACV-MVA (B16 in CPXV) [70], as well as EVM002, EVM005, and EVM154 of ECTV (D3/H3, D8, and B3 in CPXV) [71] that they interact with the cellular SCF complex (Figure 1(a)) and, presumably, provide for specific interaction with substrate proteins of cellular or viral origins, which are then ubiquitinated by this complex. An important problem is to detect these substrate proteins for each of the numerous orthopoxvirus proteins of the considered family.

### 3.2. Viral BTB-Kelch-Like Proteins

Among all the viruses, only the representatives of the family Poxviridae contain the genes of kelch-like proteins in their genomes. According to structural similarity, they are ascribed to the same group as *Drosophila *kelch protein (BTB-kelch) [72–74]. These proteins contain the N-terminal BTB domain and C-terminal kelch domain. Computer analysis of orthopoxvirus genomes has demonstrated that CPXV codes for six BTB-kelch family proteins with a size of about 500 AA residues each and mutual identity in AA sequence in the range of 22–26%. VACV genome codes for only three full-sized kelch-like proteins, which are highly homologous to the corresponding CPXV proteins; as for the highly attenuated strain VACV-MVA, unable to replicate in the majority of mammalian cell lines, it retained only one gene of this family [65]. The same gene is the only gene in MPXV genome encoding a BTB-kelch protein. As for VARV genome, all genes of this family are destroyed due to multiple mutations; consequently, only short potential ORFs, which are nonfunctional fragments of the genes of a precursor virus, are detectable in this virus [74] (Table 3). ECTV codes for four genes of the considered family—*EVM18*, *EVM27*, *EVM150*, and *EVM167*, which correspond to the CPXV-GRI genes *C18L*, *G3L*, *A57R*, and *B19R* [23, 75].

The absence of the genes from this family in various VARV isolates and the possibility of their deleting in VACV without any loss in its viability in cell culture [76] indicate that these genes are not vitally important for orthopoxvirus replication in cultured cells. Presumably, these genes are important for manifestation of species-specific properties of orthopoxviruses *in vivo*. It has been assumed that these genes can play a role in adaptation, that is, they can determine the host range (tissue tropism) and/or the possibility of virus persistence in animal body [23]. In particular, CPXV, low pathogenic for humans and displaying the widest range of sensitive animals in nature, codes for the largest set of BTB-kelch proteins. In VARV, highly pathogenic for its only host, human, in organism of which this virus cannot persist, all the genes of BTB-kelch subfamily are mutationally destroyed [63, 64].

The available data suggest that various BTB-kelch proteins interact with CUL3 (Figure 1(b)) rather than with the other cullins, that is, BTB-kelch proteins are substrate-specific adaptors for CUL3 ubiquitin-ligase complex and regulate modification and/or degradation of various proteins [42].

When studying the properties of orthopoxvirus BTB-kelch proteins, it has been found that the ECTV proteins EVM150 and EVM167 are involved in formation of active CUL3-containing ubiquitin ligases [77]. Two other proteins, EVM18 and EVM27, also interact with CUL3 [78]. Since the mutual homology of these viral proteins is low, it is likely that their functions are different and they interact with different targets. It has been experimentally demonstrated that a directed deletion of individual genes encoding EVM18, EVM27, or EVM167 radically decreases the ECTV virulence for white mice, while the damage of EVM150 gene has no effect on the virulence [79].

Deletion of four CPXV* BTB-kelch* genes led to a decrease in the cytopathic effect on cell culture and statistically significant reduction in formation of the virus-induced cytoplasmic pseudopodia [80, 81].

Deletion of individual *kelch*-like genes in the VACV genome provided for demonstrating that the damage of genes *C2L* or *A55R* (see Table 2) led to similar effects, appearing as changes in the morphology of virus plaques on cell culture monolayer, decrease in virus-induced cytoplasmic pseudopodia, decrease in Ca^2+^-independent adhesion of VACV-infected cells, and induction of larger lesions in the model of intradermal infection of mouse ear pinnae as compared with the wildtype virus [82, 83]. Damage of the VACV *kelch*-like gene *F3L* did not cause so pronounced effects [84]. Interestingly, this particular single *BTB-kelch* gene remained in MPXV (see Table 3) and the highly attenuated VACV strain MVA.

The ability of orthopoxvirus BTB-kelch-like proteins to interact with Cullin-3-containing ubiquitin-protein ligase to a considerable degree relates this family to the family of orthopoxvirus ankyrin-F-box-like proteins interacting with Cullin-1-containing ubiquitin-protein ligase. Most likely, the proteins of these two families are involved in organization of the multifactorial intricate system of interactions of virus proteins with one another and cellular components. We believe that such interactions can determine a wide range of animal tissues and species sensitive to CPXV as well as for the tolerant mode of relationships between this virus and the host. One can speculate that destruction of the majority of the genes/proteins belonging to these two families, characteristic of VARV, is the most likely reason underlying a drastic narrowing of the VARV host range and its transition to an “aggressor” mode [63]. Note that VARV retained five genes encoding ankyrin-F-box proteins (Table 2), whereas the genes for BTB-kelch proteins are completely destroyed (Table 3).

## 4. Viral Apoptosis Inhibitors

One of the first lines of the organism's nonspecific defense against infectious agents and probably one of the most ancient ones is apoptosis (programmed cell death) [85, 86]. After a cell has been infected by a virus, apoptosis serves to kill the cell, thus preventing virus proliferation and protecting nearby cells from the infection. Apoptosis is a very common phenomenon in multicellular organisms. It is primarily mediated by cysteine proteases termed caspases. An important role in apoptosis regulation belongs to mitochondria and Bcl-2 proteins. Interferon-induced synthesis of RNase L (see Section 5) causes apoptosis mediated by caspases 8, 9, and 2 [87].

A key function in the induction of programmed cell death is performed by cellular caspase 1, which specifically cleaves inactive prointerleukin-1*β* and producing the mature interleukin-1*β* form (IL-1*β*). It should be noted that IL-1*β* itself is not associated with apoptosis; that is, it is other proteins that caspase-1 targets when triggering programmed cell death [88]. It was revealed that the product of the CPXV gene *SPI-2* is an inhibitor of caspases 1 and 8 and, therefore, an apoptosis inhibitor [89, 90]. A comparison of SPI-2 amino acid sequences showed that CPXV and VACV proteins are very similar but differ significantly from the VARV isolog [3].

Based on an *in silico* amino acid sequence analysis, another VACV protein, C12 (in VARV-IND, B25; see Table 4), was also classified into the same family of protease inhibitors as SPI-2 and named SPI-1; it was shown to act as an apoptosis inhibitor, too. However, the principle of its action so far remains unclear. Supposedly, SPI-1 inhibits a caspase-independent apoptosis pathway [91].

VACV protein F1 is localized in mitochondria and acts as a caspase 9 inhibitor, suppressing the programmed death of infected cells [92–94]. The amino acid sequence of another conserved orthopoxvirus protein, N1 (VACV-COP, Table 4), has little homology to the Bcl-2 sequence, but its tertiary structure closely resembles the proteins of this family, and N1 is an apoptosis inhibitor (see additionally Section 2) [34, 95].

Some VACV strains, as well as the camelpox virus, contain a gene of a transmembrane protein of 237 amino acids located in the Golgi apparatus, which suppresses apoptosis of infected cells [96]. CPXV encodes a somewhat shorter version of the same protein (T1), while VARV and MPXV lack gene of the isolog protein (Table 4).

Double-stranded RNA apparently also can induce apoptosis, as suggested by investigation of a VACV strain carrying a mutant *E3L* variant. It was shown that disruption of this gene results not only in increased interferon sensitivity of the virus (see Section 5) but also in activation of apoptosis of infected cells [97]. This gene is fairly well conserved among different orthopoxvirus species.

The first orthopoxvirus ubiquitin ligase belonging to the family of mono-subunit RING-containing E3 ligases was found in ECTV. First, it was shown that the RING domain-containing ECTV protein p28 is a virulence factor inhibiting TNF-induced apoptosis [98]. This viral protein localized in cytoplasmic virus factories is essential for virus replication in macrophages [99]. Under consideration of the growing body of knowledge concerning RING domain-containing ubiquitin ligases, investigation of ECTV and VARV p28 proteins showed that p28 acts as an ubiquitin ligase [100]. The respective gene is highly conserved among VARV, MPXV, CPXV, and ECTV, but inactivated in the known VACV strains. The molecular target (viral or cellular protein) of orthopoxvirus ubiquitin ligase p28 has not been identified yet.

Thus, orthopoxviruses possess at least seven genes encoding apoptosis inhibitors with very diverse modes of action (Table 4). This observation further confirms the importance of apoptosis in the mammalian system of antiviral defense.

## 5. Viral Interferon Inhibitors

Mammalian cells respond to viral infection by producing interferons (IFNs). The initial production of type I IFNs is due to activation of IFN regulatory factors (IRFs), and in particular IRF3, downstream of PRRs, which recognize viral DNA, RNA, and proteins [33]. It was discovered that some orthopoxviral Bcl-2-like proteins inhibit PRR-induced activation of IRFs (see Section 2 and Table 1) and therefore suppress IFN production.

IFNs are produced and secreted by animal cells also in response to double-stranded RNA molecules (dsRNA) synthesized in the course of viral infection. IFNs bind to specific cell receptors and induce antiviral defense state [101]. IFN-induced antiviral cell state is determined by at least two enzymatic pathways. One of them involves IFN-induced dsRNA-activated protein kinase (PKR); another one depends on 2-5A[ppp(A2′p)nA] synthetase (usually termed 2-5A synthetase). The protein kinase is activated by autophosphorylation, which occurs after protein binding to dsRNA. The activated PKR phosphorylates subunit alpha of the eukaryotic translation initiation factor (eIF-2*α*), thus blocking protein synthesis. The other enzyme, 2-5A synthetase is activated by dsRNA and catalyzes ATP polymerization to 2′-5′oligoadenylates, which, in turn, activate latent cellular endo-RNase L. RNase L cleaves mRNA and rRNA molecules, thus also disturbing protein synthesis. 

Although orthopoxviruses produce high levels of virus-specific dsRNA in the late phase of their life cycle [102], they are highly resistant to IFN action [103]. VACV gene *E3L* encodes an inhibitor of the IFN-induced PRK [104]. This viral protein produced directly after cell infection can bind to dsRNA, competing with the specific cellular protein kinase and preventing the enzyme activation. Another VACV gene, *K3L*, encodes an eIF-2*α* homologue competing with endogenous eIF-2*α* for phosphorylation by activated PRK [105]. A mutant VACV strain with disrupted *K3L* is interferonsensitive and produces two orders of magnitude less viral progeny [106]. Thus, orthopoxviruses produce proteins that inhibit the activity of the IFN-induced PRK in two independent ways. 

It should be noted that although the sequences of viral eIF-2*α* homologues are well conserved within species, the VARV protein has numerous differences in amino acids from highly homologous VACV and CPXV isologs, while MPXV does not encode this interferon resistance factor due to multiple mutations of the respective gene (Table 4).

In the course of VACV infection, *E3L* is expressed from the first and the second initiator codon producing the long and the short protein forms, respectively [106]. The N-terminal domain of the long form is required for the protein binding to Z-form DNA, which explains its nuclear localization and its pathogenic properties [107, 108]. The C-terminal domain of both the long and the short form binds dsRNA and inhibits the activation of the PRK [109] and 2-5A synthetase [110]. In MPXV-ZAI, the first initiator triplet is disrupted by a mutation; for this reason, only the short protein form is translated. Thus, MPXV differs from other orthopoxvirus species in the unique organization of viral intracellular interferon resistance factors [64, 111], which apparently results in decreased propagation rates *in vivo* and, consequently, decreased efficiency of airborne transmission of the virus, which is indeed the case for human monkeypox, as compared to smallpox.

Recently, it has been demonstrated that VACV E3 protein also inhibits the type III *λ*-IFN-mediated antiviral response [112].

Blocking the function of Stat (signal transducer and activator of transcription) proteins, which are critical for antiviral responses, has evolved as a common mechanism for pathogen immune evasion. The VACV-encoded phosphatase H1 is critical for virus replication and plays an additional role in the evasion of host defense by dephosphorylating Stat1 and blocking IFN-stimulated innate immune responses. It was demonstrated that VARV H1 isolog (I1 for VARV-IND, see Table 4) is more active than VACV H1 in Stat1 dephosphorylation [113].

A unique and efficient IFN evasion strategy employed additionally by poxviruses is to encode soluble proteins that are secreted from infected cells and function as soluble IFN decoy receptors. These factors providing interferon resistance in orthopoxviruses are extracellular *γ*-IFN-binding protein [114] and type I *α*/*β*-IFN-binding protein (*α*/*β*-IFN-BP) [115]. Earlier we have revealed pronounced species-specific differences in amino acid sequences of *α*/*β*-IFN-BPs of orthopoxviruses [3, 116]. Recently it has been shown that the VARV *α*/*β*-IFN-BP binds the human ligands with higher affinity than the VACV *α*/*β*-IFN-BP [117].

Thus, orthopoxviruses possess a multigene system providing a high level of interferon resistance. Differences found among these genes/proteins in VARV, MPXV, CPXV, and VACV (Table 4) call for further investigation of their properties.

## 6. Viral Inhibitors of Inflammatory Response

Inflammatory reactions play an important role in the early nonspecific protection of the organism against the viral infection. They are induced rapidly to limit the virus dissemination during the first hours and days upon infection while the full-fledged adaptive immune response is being formed. It is known that the complement system and the cytokines, such as tumor necrosis factor (TNF), interleukin-1*β* (IL-1*β*), gamma-interferon (*γ*-IFN), and chemokines, play the key role in inducing the inflammatory reactions [118]. In addition, several other mediators influence either directly or indirectly the development of the inflammatory process [116, 119–121]. Therefore, poxviruses potentially need several genes whose protein products are able to act as inhibitors of various stages of inflammation development to suppress efficiently the inflammatory response.

The first viral gene whose product represses inflammatory response to infection was found in CPXV and termed SPI-2 (*B12R* for CPXV-GRI, see Table 4) [122]. As noted above (see Section 3), SPI-2 inhibits caspase 1 activity and thus prevents the processing of pro-IL-1*β* to IL-1*β* and its secretion from the infected cell, suppressing, as a result, the induction of local inflammatory reactions. In addition, SPI-2 inhibits the production of inflammatory mediators (leukotrienes) in the arachidonic acid metabolism [123]. Furthermore, as discussed above, SPI-2 is also involved in suppressing apoptosis of the infected cell. Thus, this protein evidently plays an important role in determining orthopoxvirus pathogenicity *in vivo*. It should be noted that amino acid sequences of SPI-2 variants present in VARV, MPXV, and CPXV are somewhat different [32]. In the case of VACV-COP, the gene encoding this protein is damaged (Table 4).

It was shown experimentally that the VACV-WR gene *B15R* encodes a secreted glycoprotein acting as a soluble IL-1*β* receptor [124]. The production of this soluble receptor prevents the development of systemic reactions (such as fever) in VACV-infected mice [125]. It was shown that a VACV-WR strain with disrupted *B15R* had increased virulence in mice (when administered intranasally) [124]. A further analysis showed that VACV strains associated with a higher frequency of postvaccination complications in humans lack IL-1*β*-binding activity [125]. These data agree well with the fact that the respective gene in VARV is disrupted (by fragmentation) (Table 4). 

Thus, we may hypothesize that VARV suppresses production and secretion of IL-1*β* by infected cells but does not inhibit the effect of extracellular IL-1*β* synthesized by other cells of the body. This suggests that VARV is capable of suppressing local inflammatory reactions due to SPI-2 production in the region of virus replication; however, it does not inhibit the systemic reactions, as it is unable to synthesize IL-1*β*-binding protein. Decrease in the local inflammatory reactions may assist a more active virus replication, while uncontrolled development of the systemic reactions weakens the overall resistance of the organism to infection. A concurrent development of these reactions is likely to boost the pathogenic effect of the viral infection on the host organism. In the case of MPXV and CPXV, both genes in question are native (Table 4).

Orthopoxviruses, in particular, VACV-WR, but not the less virulent VACV-COP strain, also encode an IL-18-binding protein (Table 4), which is secreted from the cell and suppresses the activity of proinflammatory IL-18 [126].

Similarly to other cytokines, TNF performs multiple functions [118]. In particular, as noted above, it is a key cytokine inducing inflammation in the infected host along with IL-1*β* and IL-18. It was shown that VARV-IND gene *G2R* encodes CrmB protein homologous to type II TNF receptor [127]. An orthologous TNF-inhibitory protein called M-T2 is an important secreted virulence factor of the rabbit myxoma virus (a poxvirus of the genus *Leporipoxvirus*). Its VARV analogue G2 apparently has similar properties. An important difference between VARV and VACV is that the latter possesses no genes encoding TNF receptor analogues. In the CPXV genome, we detected five genes of the TNF receptor family [23]. Four of them have TNF-binding activity [128–131] (Table 4). 

An analysis of amino acid sequences of CrmB isologs detected numerous species-specific differences. Using a baculuvirus expression system, we obtained individual CrmB proteins of VARV, MPXV, and CPXV and showed that their ability to suppress the activity of human, mouse, and rabbit TNFs differs considerably. Only CrmB-VARV inhibits human TNF activity with high efficiency [132, 133]. Presumably, this is a result of evolutionary adaptation of the viral receptors to the ligands of their hosts. 

It was recently shown that orthopoxvirus TNF-binding protein CrmB possesses a further biological activity; that is, it has high affinity to certain chemokines critically involved in attracting dendritic cells, B-, and T-lymphocytes to the inflammation focus [134]. Its immunomodulatory activity is determined by the unique C-terminal domain termed SECRET (smallpox virus-encoded chemokine receptor). The amino acid sequence of this domain has no homology to any vertebrate protein or to any other known viral chemokine-binding protein. *De novo* modeling of the spatial structure of the SECRET domain showed that it might be a structural homologue of the secreted CC-chemokine-binding protein G3 of VARV (Table 4), in spite of the low similarity of their amino acid sequences [135].

Chemokines are chemoattractant cytokines, which control migration and effector functions of leukocytes, thereby playing an important role in development of inflammatory response and protection against pathogens [136]. It was demonstrated that VACV strain Lister at the early stages of infection produced a protein secreted from the cells in large amounts [137], which bound a wide range of CC chemokines and inhibited their activities [138]. This gene is damaged in many other VACV strains. Presumably, isologs of this protein (G3 in VARV-IND) of various orthopoxvirus species have different functions, as analysis of their amino acid sequences detected considerable species-specific distinctions [3]. 

The VACV protein A41 and its orthopoxvirus isologs also are secreted glycoproteins that efficiently and selectively bind to certain CC and CXC chemokines preventing chemokine-induced leukocyte migration to the infection locus [139, 140]. This chemokine-binding protein probably is essential for virus propagation, since it is conserved in all orthopoxvirus species studied (Table 4). 

Interestingly, all orthopoxviruses in question also produce a soluble *γ*-IFN-receptor, which can modulate the host's inflammatory response to infection [141–143]. The protein B9 of VARV-IND and its isolog produced by VACV-COP contain a considerable number of amino acid substitutions [144]. Probably, these species-specific differences in the structure of viral *γ*-IFN-binding protein are related to the difference in VARV and VACV virulence. 

In addition to the above genes, orthopoxviruses also carry a gene of a complement-binding protein (*C3L* in VACV-COP) [145], one of whose functions may be regulation of inflammation. The complement system comprises over 20 blood plasma proteins. Antiviral functions of the complement systems include virus neutralization, lysis of infected cells, and enhancement of inflammatory and adaptive immune response [2, 119]. 

The VACV protein C3, named VCP, secreted from infected cells and controlling the reaction of complement activation comprises four short degenerated repeats of approximately 60 amino acids each (short consensus repeat, SCR) characteristic of the protein family of complement activation regulators (RCA) [146]. It is considered that the gene encoding VCP originated initially due to incorporating a part or the complete coding sequence of a protein belonging to RCA family of the host into the viral genome followed by adaptation (alteration) of the gene in question to perform the functions necessary for the virus [147]. The X-ray structural analysis showed that SCR sequences of VCP form a series of discrete tightly linked compact domains [148].

VCP is a unique multifunctional viral protein functionally resembling as different RCA proteins as factor H, membrane-bound cofactor protein, type I complement receptor, and decay-accelerating factor (DAF). Firstly, VCP binds complement components C3b and C4b; secondly, it blocks different stages of the complement cascade and inhibits both the classical and the alternative complement pathways; thirdly, it blocks complement-driven virus neutralization activated by antiviral antibodies, and, finally, binds heparin-like molecules on the surface of endothelial cells, blocking the binding of chemokines and preventing signal transduction for chemotaxis [119]. The model of CPXV-infected mice showed that VCP suppresses inflammatory response *in vivo* [149, 150].

VCPs of VARV, CPXV, and VACV contain four SCRs each. We have revealed the unique structure of the MPXV VCP [3, 151]. Due to premature termination of synthesis, the protein sequence is truncated and the C-terminal SCR-4 is deleted in Central African MPXV strains, whereas Western African MPXV strains lack the gene for VCP completely [3]. Possibly this deletion or truncation of the gene for VCP prevents effective inhibition of inflammatory response by MPXV and therefore the specific feature of human monkeypox clinical course, distinguishing it from smallpox, is lymphadenitides.

Amino acid sequences of VACV and VARV VCPs differ at 12 positions. A baculovirus system was used to produce individual VCPs of VARV and VACV [152]. It was shown that VCP of VARV is a significantly more efficient inhibitor of human complement than its VACV counterpart. This observation further supports the concept that viral soluble receptors are evolutionary adapted to the host's ligands.

To sum up, orthopoxviruses possess a multigene system controlling at different stages development of the host's inflammatory reactions. Orthopoxviruses display species-specific distinctions not only in the set of these genes but also in their structure, and as a result in targeted activities of the encoding proteins.

## 7. Orthopoxvirus Modulation of Cellular ****Immune Response

One of the principal mechanisms of the innate cellular immunity involves nonspecific lysis of virus-infected cells by natural killer (NK) cells [153]. The latter are activated by soluble mediators or in a direct cell contact. NK proliferation peaks on days 2-3 of orthopoxvirus infection; however, these cells alone are unable to prevent completely the dissemination of infection in the body [154]. NK cell activation is regulated by integrated signals of several activating and suppressing receptors, many of which use major histocompatibility complex (MHC) class I molecules or related proteins as ligands. One of the cytotoxic NK-activating receptors is NKG2D. It was shown that CPXV and MPXV encode a protein resembling an MHC class I molecule (OMCP; C2 in CPXV-GRI) that blocks the recognition of host ligands and inhibits the NKG2D-dependent NK lysis of infected cells [155]. VARV and VACV genomes lack such a gene (Table 4).

As noted above, orthopoxviruses produce a secreted IL-18-binding protein, which blocks not only the proinflammatory activity of IL-18 but also IL-18 induced NK cytotoxicity [126, 156].

Adaptive immune response to infection involves complex cytokine-regulated interactions among different types of cells [157] that give rise to B-lymphocytes producing virus-specific antibodies and virus-specific cytolytic T lymphocytes. Proliferation of virus-specific cytolytic T lymphocytes peaks on days 5-6 of infection; the key regulatory cytokines TNF, IL-1*β*, and *γ*-IFN control not only inflammatory reactions but also the adaptive immune response. Specific antibodies can interact with virus particles and their components individually or within complement complexes. Specific antibodies are inefficient in controlling primary poxvirus infection but can be important in preventing secondary infection [154]. Cellular immune response is a crucial component of specific defense against a poxvirus infection [158]. 

For some orthopoxviruses, it has been shown that they directly infect human and rodent immune cells both *in vitro* and *in vivo*, including lymphocytes, NK cells, and monocytes/macrophages and VACV decrease antigen presentation in several types of antigen-presenting cells (APC) [159]. Recently, it was revealed that VACV-COP protein A35 inhibits MHC class II-restricted antigen presentation, immune priming of T lymphocytes, and subsequent cytokine and chemokine synthesis [160]. The gene of this protein is highly conservative for VACV, CPXV, and MPXV but in the genome of VARV is disrupted (see Table 4). Interestingly, for VACV MVA strain it was shown that deletion of the *A35R* gene increases its immunogenicity [161]. In this respect, it should be noted known data that the persons that had smallpox acquire a lifelong immunity, whereas the vaccination with VACV requires repeated immunizations with a certain periodicity to provide a reliable protection against smallpox [10]. We may speculate that VARV nonfunctional short *A38R* originated as a result of mutational changes in functional *A35R* analog of ancestral zoonotic orthopoxvirus and it allowed to highly virulent VARV caused lifelong immunity in humans and provided the additional conditions for smallpox endemization [4].

MHC class I molecules play an important role in antiviral immunity. The majority of MHC class I-binding peptides are generated in the cytosol by proteasomes and transported into the lumen of the endoplasmic reticulum (ER) by transporter associated with antigen processing (TAP). Peptide loading onto the MHC class I heavy chain-*β*2 m heterodimer is facilitated by a multi-subunit protein complex called the MHC class I peptide-loading complex (PLC). In addition to TAP and MHC class I, the PLC is composed of ER chaperones. Upon peptide loading, the fully assembled MHC class I complexes dissociate from the PLC and transit to the cell surface. Recognition of viral peptides in the context of MHC class I molecules triggers virus-specific CD8 T cells to exert their effector functions including cytotoxicity and cytokine secretion [162]. 

Recently, it has been shown that CPXV downregulates MHC class I and evades antiviral CD8 T cell responses [163]. Two distinctly acting MHC class I regulating genes (*D10L* and *B8R* for CPXV-GRI, see Table 4) have been revealed. Protein D10 inhibits MHC class I expression by impairing ER peptide loading and dissociation of MHC class I from TAP. Protein B8 interferes with the intracellular trafficking of MHC class I molecules by sequestering them in the ER using its C-terminal KDEL-like sequence [162]. 

The *in vivo* significance of the discovered viral MHC class I and class II evasion mechanisms, however, is not well understood. Among orthopoxviruses pathogenic for humans only CPXV produces all three known regulators of MHC systems. Highly virulent and highly immunogenic for humans, VARV does not produce any of these proteins (Table 4).

## 8. Conclusion

Comparison of amino acid sequences of a great number of various types of human and rodent polypeptides revealed most pronounced interspecies differences in the sequences of the proteins forming ligand-receptor pairs of the organismal protective systems of these mammals against infectious agents. In addition, the polypeptide ligands and their receptors proved to be subjected to coevolution [164]. Pathogenic microorganisms are assumed to be able to cause an accelerated evolution of the defense system proteins (genes) of infected animal species. Such evolutionary changes in the primary structure of the proteins constituting ligand-receptor pairs were suggested to result in alterations of the quaternary structure of the ligand-receptor contact region [164, 165]. As a result, the species-specific mimicry of mammalian defense system proteins may emerge, providing a narrower range of hosts sensitive to certain infectious microorganism [164].

The virus that maintains the balance between its pathogenic effect on the host organism and the possibility of its effective development in the animal organism for a relatively long period is the best adapted from the evolutionary standpoint. Such virus is able to transmit efficiently from animal to animal under a low population density. Among orthopoxviruses, CPXV most pronouncedly displays such properties. It is worth noting that CPXV encodes the complete set of immunomodulatory (Table 4) as well as Bcl-2-like (Table 1), ankyrin-like (Table 2), and kelch-like (Table 3) proteins found in orthopoxviruses, whereas VARV, MPXV, and VACV each possess an incomplete species-specific subset of these genes. 

The species- and strain-specific distinctions between VARV, MPXV, CPXV, and VACV DNAs are localized to the long variable terminal regions [3, 23, 63, 64, 166–168]. These distinctions comprise not only deletions in DNAs of the viruses compared relative one another but also rearrangements and nucleotide substitutions [3, 169]. The determined sequences of viral DNAs allow for a comparative analysis of organization of the VARV, MPXV, CPXV, and VACV molecular pathogenicity factors, whose function was verified at various laboratories in experiments mainly with VACV, CPXV, and ECTV [3, 22, 56, 116, 120, 121, 154, 157].

The CPXV genome has the largest size as compared with the other orthopoxviruses and contains the complete set of all the genes characteristic of other viruses from the genus *Orthopoxvirus* (Tables 1, 2, 3, and 4). The fact that CPXV nonetheless does not display an increased virulence suggests that orthopoxviruses have a certain regulatory system(s). To describe this regulatory system, we earlier introduced the concept of *buffer* genes, whose role is to neutralize the negative effects developing in the body during infection [22]. Presumably, CPXV possesses the widest set of these genes as compared with VARV, MPXV, and VACV. The rest orthopoxvirus species have lost certain part of the genes with reference to CPXV. Note that VARV contains the shortest orthopoxviral genome and the least set of actual genes. This observation suggests that CPXV is most close to the ancestor of orthopoxviruses, while the rest species emerged later due to deletions, recombinations, and mutations [3, 5, 6, 169].

Humans are the only VARV host; hence, this virus is to a maximal degree adapted evolutionarily to overcome the human defense reactions, which develop in response to the infection. MPXV, CPXV, and VACV have wide host range, infecting first and foremost various rodents. Humans are sporadically infected by these viruses. Consequently, MPXV, CPXV, and VACV are adapted to interactions with the molecular defense reactions of mammals of various species [3, 4, 11, 13, 19, 21]. As we can see from the above-mentioned in these review cases, studied immunomodulatory proteins of VARV, as a rule, more effectively inhibit activities of their human ligands as compared with other species of orthopoxviruses pathogenic for humans. We may speculate that it is one of the most likely reasons of high VARV virulence for humans.

The interaction network of cytokines and their receptors has been so far studied only to a first approximation, and many discoveries are still awaiting researchers in this direction. Orthopoxviruses can play an important role here. 

Summing up the available data, we may infer that VARV and other orthopoxviruses possess an unexampled set of genes whose protein products efficiently modulate the manifold defense functions of the host organisms compared with the viruses from other families. It is likely that by the example of orthopoxviruses, it will be possible in the nearest future to trace the patterns of coevolution of the viral pathogenicity factors and mammalian systems providing defense against infectious agents. The research into application of immunomodulatory proteins of orthopoxviruses, and first and foremost, variola virus, as drugs also deserves attention.

## Figures and Tables

**Table 1:** Orthopoxviral Bcl-2-like proteins.

Protein function	VACV-COP		CPXV-GRI		MPXV-ZAI		VARV-IND	
	ORF	Size, aa	ORF	Size, aa	ORF	Size, aa	ORF	Size, aa
Inhibition of NF-*κ*B and IRF3 activation by interacting with MyD88, TIRAP and TRIF, and TRAM	A46R	214	A49R	240	A47R	240	A52R	240
Inhibition of NF-*κ*B activation by interacting with IRAK2 and TRAF6	A52R	190	A55R	190	—	—	J6R	71
Inhibition of NF-*κ*B activation by interacting with IKK complex	B15R	149	B13R	149	B13R	149	B14R	149
Inhibition of NF-*κ*B and IRF3 activation by interacting with IKK complex and TBK1, apoptosis inhibitor	N1L	117	Q1L	117	P1L	117	P1L	117
Inhibition of NF-*κ*B and IRF3 activation by interacting with IRAK2, TRAF6, and DDX3	K7R	149	M6R	149	C6R	149	C4R	149
Inhibition of IRF3 and IRF7 activation by interacting with TANK, NAP1, and SINTBAD	C6L	151	C14L	156	D11L	153	D9L	156
Unknown	C1L	224	C19L	231	D19L	214	D14L	214
Unknown	C16L	181	D5L	153	—	—	D1L	153
Unknown	N2L	175	Q2L	175	P2L	177	P2L	177

VARV-IND: VARV strain India,1967, MPXV-ZAI: MPXV strain Zaire-I-96, CPXV-GRI: CPXV strain GRI-90, VAC-COP: VACV strain Copenhagen. ORF: open reading frame; aa: protein size in number of amino acid residues.

**Table 2:** Orthopoxviral ankyrin-F-box-like proteins.

VACV-COP		CPXV-GRI		MPXV-ZAI		VARV-IND	
ORF	Size, aa	ORF	Size, aa	ORF	Size, aa	ORF	Size, aa
C19L^∗^	259	***D3L*** ^ ∗^	**586**	**J3L** ^ ∗^	**587**	None	None
C17L^∗^	386	**D4L** ^ ∗^	**672**	None	None	None	None
None	None	***D8L***	**661**	None	None	None	None
None	None	**D14L**	**764**	None	None	None	None
None	None	**C1L**	**437**	**D1L** ^ ∗^	**437**	None	None
None	None	**C3L**	**833**	None	None	None	None
None	None	***C9L***	**668**	**D7L**	**660**	D6L	452
**C9L**	**634**	**C11L**	**614**	**D9L**	**630**	D7L	153
**M1L**	**472**	**O1L**	**474**	**O1L**	**442**	**O1L**	**446**
K1L	284	M1L	284	C1L	284	C1L	66
**B4R**	**558**	***B3R***	**558**	**B5R**	**561**	**B6R**	**558**
**B18R**	**574**	***B16R***	**574**	None	None	**B19R**	**574**
B20R	127	**B18R**	**795**	**B17R**	**793**	**B21R**	**787**
B21R^∗^	91	**K1R**	**581**	None	None	None	None
None	None	None	None	**N4R** ^ ∗^	**437**	None	None
B23R^∗^	386	**H2R** ^ ∗^	**672**	None	None	None	None
B25R^∗^	259	***H3R*** ^ ∗^	**586**	**J1R** ^ ∗^	**587**	**G1R**	**585**

Asterisks denote ORFs that are duplicated in left and right inverted terminal repeat regions of the viral genome. ORFs with full length are set in bold. The ORFs for the proteins with experimentally confirmed interaction with the cellular Cullin1-containing ubiquitin-protein ligase are indicated by bold italic letters.

**Table 3:** Orthopoxviral BTB-kelch-like proteins.

VACV-COP		CPXV-GRI		MPXV-ZAI		VARV-IND	
ORF	Size, aa	ORF	Size, aa	ORF	Size, aa	ORF	Size, aa
None	None	**D11L**	**521**	None	None	None	None
**C2L**	**512**	***C18L***	**512**	D18L	107	None	None
**F3L**	**480**	***G3L***	**485**	**C9L**	**487**	C7L	179
**A55R**	**564**	***A57R***	**564**	None	None	J7R	71
B10R	166	**B9R**	**501**	None	None	None	None
None	None	***B19R***	**557**	B18R	70	B22R	70

ORFs with full length are set in bold. The ORFs for the proteins with experimentally confirmed interaction with the cellular Cullin 3-containing ubiquitin-protein ligase are indicated by bold italic letters.

**Table 4:** Orthopoxviral proteins modulating defense reactions of mammals.

Protein function	VACV-COP		CPXV-GRI		MPXV-ZAI		VARV-IND	
	ORF	Size, aa	ORF	Size, aa	ORF	Size, aa	ORF	Size, aa
Apoptosis inhibitor, caspase-1 and caspase-8 inhibitor, SPI-2	**B13R**	**116**	B12R	345	B12R	344	B13R	344
Apoptosis inhibitor, SPI-1	C12L	353	B20R	375	B19R	357	B25R	372
Apoptosis inhibitor, Bcl-2-like	N1L	117	Q1L	117	P1L	117	P1L	117
Mitochondria-associated apoptosis inhibitor, caspase-9 inhibitor	F1L	226	G1L	238	C7L	219	C5L	251
Apoptosis inhibitor, transmembrane protein of Golgi apparatus	**None**	**None**	T1R	210	**R1R**	**105**	**None**	**None**
Apoptosis inhibitor, RING-domain containing E3 ubiquitin ligase	**None**	**None**	C7R	242	D5R	242	D4R	242
Apoptosis inhibitor, dsRNA-binding, interferon resistance	E3L	190	F3L	190	**F3L**	**153**	E3L	190
eIF-2*α* homolog, interferon resistance	K3L	88	M3L	88	**None**	**None**	C3L	88
Phosphatase, dephosphorylation of Stat 1	H1L	171	J1L	171	H1L	171	I1L	171
*γ*-IFN-binding	B8R	272	B7R	271	B9R	267	B9R	266
*α*/*β*-IFN-binding	B19R	353	B17R	351	B16R	352	B20R	354
IL-1*β*-binding	**None**	**None**	C8L	124	D6L	126	D5L	126
IL-18-binding	**B16R**	**290**	B14R	326	B14R	326	**B15R**	**63**
Complement binding	C3L	263	C17L	259	**D14L**	**216**	D12L	263
TNF- and chemokine-binding, CrmB	**B28R**	**122**	H4R	351	J2R	348	G2R	349
TNF-binding, CrmC	**A53R**	**103**	A56R	186	**None**	**None**	**None**	**None**
TNF- and chemokine-binding, CrmD	**None**	**None**	K2R	322	**None**	**None**	**None**	**None**
TNF-binding, CrmE	**None**	**None**	K3R	167	**K1R**	**70**	**None**	**None**
CC-chemokine-binding	**B29R**	**244**	I5R	255	J3R	246	G3R	253
CC- *и* CXC-chemokine-binding	A41L	219	A43L	219	A41L	221	A46L	218
Inhibitor of NK-mediated NKG2D-dependent lysis of infected cells	**None**	**None**	C2L	178	N3R	176	**None**	**None**
Inhibitor of MHC class II antigen presentation	A35R	176	A36R	176	A37R	176	**A38R**	**60**
Inhibitor of MHC class I complexes release from the PLC	**None**	**None**	D10L	96	**None**	**None**	**None**	**None**
Inhibitor of the intracellular trafficking of MHC class I molecules	**B9R**	**77**	B8R	221	B10R	221	**None**	**None**

ORFs that are altered/nonfunctional as compared with a CPXV-GRI counterpart are set in bold.
